# Phonocardiogram (PCG) Murmur Detection Based on the Mean Teacher Method

**DOI:** 10.3390/s24206646

**Published:** 2024-10-15

**Authors:** Yi Luo, Zuoming Fu, Yantian Ding, Xiaojian Chen, Kai Ding

**Affiliations:** 1 Department of Biomedical Engineering, Johns Hopkins University, Baltimore, MD 21287, USA; yluo62@jh.edu (Y.L.); xchen279@jhu.edu (X.C.); 2 Department of Electrical and Computer Engineering, Cornell University, Ithaca, NY 14853, USA; zf242@cornell.edu; 3 Department of Applied Mathematics and Statistics, Johns Hopkins University, Baltimore, MD 21287, USA; yding72@jh.edu; 4 Department of Radiation Oncology and Molecular Radiation Sciences, Johns Hopkins University, Baltimore, MD 21287, USA

**Keywords:** cardiovascular disease (CVD), phonocardiogram (PCG), murmur detection, semi-supervised learning, mean teacher, non-invasive assessment, low and middle-income countries (LMICs)

## Abstract

Cardiovascular diseases (CVDs) are among the primary causes of mortality globally, highlighting the critical need for early detection to mitigate their impact. Phonocardiograms (PCGs), which record heart sounds, are essential for the non-invasive assessment of cardiac function, enabling the early identification of abnormalities such as murmurs. Particularly in underprivileged regions with high birth rates, the absence of early diagnosis poses a significant public health challenge. In pediatric populations, the analysis of PCG signals is invaluable for detecting abnormal sound waves indicative of congenital and acquired heart diseases, such as septal defects and defective cardiac valves. In the PhysioNet 2022 challenge, the murmur score is a weighted accuracy metric that reflects detection accuracy based on clinical significance. In our research, we proposed a mean teacher method tailored for murmur detection, making full use of the Phyionet2022 and Phyionet2016 PCG datasets, achieving the SOTA (State of Art) performance with a murmur score of 0.82 and an AUC score of 0.90, providing an accessible and high accuracy non-invasive early stage CVD assessment tool, especially for low and middle-income countries (LMICs).

## 1. Introduction

The leading cause of mortality worldwide are CVDs [1]. Cardiac auscultation is the most accessible method for early-stage, non-invasive assessment of CVDs, particularly in LMICs. This technique involves the analysis of heart sounds, crucial indicators of heart function, and potential abnormalities. In the realm of digital health technology, PCGs elevate this basic assessment by recording these heart sounds electronically. PCGs capture the acoustic data of the heart’s beats, which can be analyzed to detect irregular patterns such as murmurs, gallops, and rubs.

In PCG recordings, the first heart sound (S1) and the second heart sound (S2) are fundamental components. S1 is produced by the closing of the atrioventricular valves, signaling the beginning of systole. In contrast, S2 is generated by the closing of the semilunar valves, marking the end of systole and the start of diastole. Beyond these, heart murmurs detected in PCG signals provide essential clinical insights into heart malfunctions due to congenital and acquired diseases in pediatric populations [2,3]. Consequently, developing machine learning algorithms that can precisely detect murmurs from PCG signals presents a significant challenge.

One major challenge in applying deep learning techniques to cardiovascular signals is the lack of annotated data. Creating reliable diagnoses requires considerable time and the expertise of highly skilled professionals, making the process demanding and resource-intensive.

Although researchers have conducted lots of work to make full use of the labeled PCG data [4,5,6], a considerable portion of unlabeled data remain underutilized. Semi-supervised learning paradigms have demonstrated notable efficacy in leveraging unlabeled data alongside a limited subset of labeled data [7,8].

The PhysioNet 2022 [9] dataset comprises heart sound recordings collected from 1568 patients, most of whom were pediatric patients from rural Brazil. These recordings were gathered using an electronic stethoscope at four key auscultation points (aortic valve, pulmonic valve, tricuspid valve, and mitral valve). A total of 5272 recordings were made, with detailed annotations provided by cardiologists. The dataset includes demographic and clinical information such as age, sex, height, and weight, as well as clinical outcomes like heart murmurs and abnormal cardiac function. The recordings are labeled into three categories—Present, Absent, and Unknown for murmurs—providing a rich source of supervised learning data. However, only 60% of this dataset (2982 recordings) was released as the public portion for model training, which we utilized in our study.

In contrast, the PhysioNet 2016 dataset [10] was primarily collected for the classification of normal versus abnormal heart sounds and includes 4430 recordings from 1072 subjects. This dataset features recordings from a diverse population of both children and adults, gathered across various environments using different recording devices. While the primary objective was to differentiate between normal and abnormal heart function, the dataset lacks the detailed murmur annotations present in the PhysioNet 2022 dataset, making it well-suited as unlabeled data for our semi-supervised learning framework. The challenge’s training set comprises 3153 heart sound recordings from 764 patients. Notably, the high-quality 2016-e subset, which has even been used for generative tasks [11], was included in our experiments and consists of 2054 PCG recordings.

In this study, we utilized the mean teacher model that leverages the labeled PCG data from Physionet 2022 in conjunction with the unlabeled PCG data in Physionet 2016, our method exhibits a comparable performance to fully supervised models. Additionally, by incorporating unlabeled data sourced from an external dataset alongside the labeled dataset, we achieve an enhanced model performance relative to utilizing labeled data alone. Our findings underscore the utility of integrating unlabeled data through a semi-supervised framework, thereby augmenting model performance and facilitating clinical decision-making by harnessing the wealth of readily available unlabeled data.

## 2. Related Work

Computer-aided classification in PCGs has captivated researchers for over 50 years, yet it continues to present challenges. Gerbarg et al. were pioneers in this field, using a threshold-based method to tackle the problem [12]. Early approaches in heart sound murmur detection involved enhancing acoustic signals through digital stethoscopes and basic electronic filtering techniques. Subsequently, techniques like the Fourier transform were employed to break down heart sounds into individual frequency components, facilitating the identification of abnormal frequencies [13,14]. Ahlström’s work comprehensively summarized the physics, analysis, and processing of heart sounds [15]. More sophisticated approaches later integrated complex signal processing methodologies, such as spectral analysis and wavelet transformations [16,17,18]. Despite these advancements, traditional methods were limited by their dependency on high-quality acoustic signals and struggled in noisy clinical environments or with low-amplitude murmur sounds.

The rise of machine learning, especially deep learning, has transformed the landscape of murmur detection. The George B. Moody PhysioNet Challenge in 2016 [10] attracted many researchers to the field, leading to significant contributions in CVD assessment. Cristhian Potes et al. [19] achieved the highest score in the challenge, which combined the results from an AdaBoost algorithm and a convolutional neural network (CNN). The AdaBoost algorithm was trained on 124 time–frequency features, while the CNN was trained on four frequency bands extracted from the PCG signals. Subsequent research utilized methods such as ResNet [20], Mel-frequency features [21], feature fusion methods [22], CNN-LSTM networks [23,24], and transfer learning [25] have achieved even higher performance.

The George B. Moody PhysioNet Challenge in 2022 [9] provided more detailed classifications for typical diagnoses, including heart murmurs. Hui Lu et al. achieved SOTA performance in murmur detection using a lightweight CNN with wide features and Mel-spectrogram [4], while LCNN, dual Bayesian ResNet, hidden semi-Markov model, and hierarchical multi-scale convolutional neural networks were also deployed to achieve competitive performance in the Physionet 2022 challenge [5,6,26,27]. Derivative works include the use of a new wav2vec model [28], multi-scale CNN with LSTM [29], and complex-valued networks [30]. Beyond the pursuit of higher classification performance, efforts have also been made to enhance model interpretability. For instance, M. Rohr et al. employed a multiple-instance learning framework to facilitate model interpretability [31], while G. Zhou et al. explored different data augmentation methods for PCG classification [32].

In the realm of semi-supervised methods, Bachman et al. [33] introduced the concept of “Pseudo-Ensembles” in 2014, which created an ensemble of models by perturbing the data and model parameters during training. This approach aims to enhance diversity in the learned representations. Further advancements were made by M Sajjadi et al. [34] in 2016, who introduced randomness into the training process to promote smoothness in the model’s predictions and increase robustness to variations in the input data [35]. Building upon this, Laine and Valpola proposed “Temporal Ensembling” in 2016, where the model’s predictions on unlabeled data were averaged over time to provide consistent pseudo-labels for regularization. These developments culminated in the introduction of the mean teacher concept by Tarvainen and Valpola in 2017 [8]. The mean teacher approach enhanced consistency regularization by encouraging the model to maintain stable predictions even when input data were slightly altered. This approach leverages both labeled and unlabeled data by comparing the student model’s current predictions with those of the teacher model, enabling simultaneous learning from both data types. This strategy greatly boosts the model’s ability to generalize and perform well on unseen data.

Despite the success of semi-supervised learning in various domains, its application in PCG murmur detection remains largely unexplored. The complexities of heart sound and the scarcity of labeled data pose significant challenges for traditional semi-supervised methods. However, integrating semi-supervised learning into PCG murmur detection could potentially alleviate the need for large labeled datasets and further improve classification performance in real-world clinical settings.

## 3. Method

Our method comprises two main components: the mean teacher model and a lightweight CNN architecture that serves as the base model.

**Figure 1:**
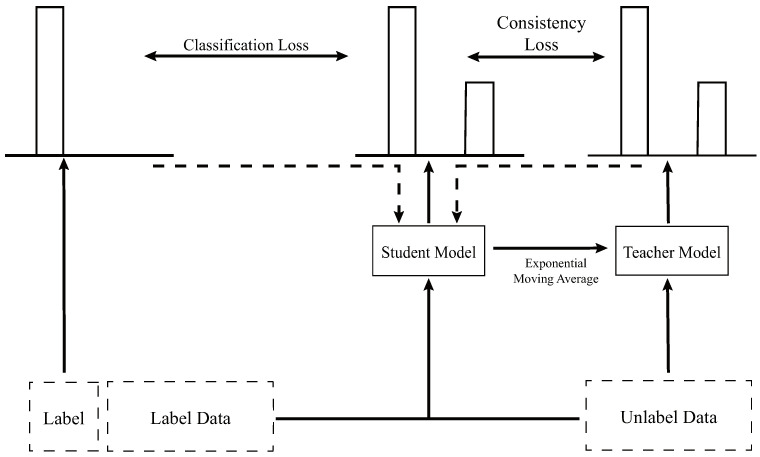
Architecture of Mean teacher model.

The mean teacher method is a semi-supervised learning strategy that harnesses unlabeled data to significantly improve model generalization and robustness [8]. Compared to traditional semi-supervised learning approaches, the mean teacher approach employs a teacher model to enhance supervision, thereby reducing the generalization error and improving the model performance; the architecture of the mean teacher model is shown in Figure 1.

In the training process of the mean teacher method, student and teacher models both utilize the same architectural framework. During each iteration, a batch of labeled data is fed into the student model, generating predictions $p_{s;1}$. Unlabeled data are also input into the student model and subsequently into the teacher model, producing predictions $p_{s;2}$ and $p_{t;2}$, respectively. The classification loss between $p_{s;1}$ and the true labels, as well as the consistency loss between $p_{s;2}$ and $p_{t;2}$, are then calculated. These losses are weighted and combined to compute the total loss, which is used to optimize the student model, as indicated by the dotted line in Figure 1. Finally, the teacher model is updated through the exponential moving average according to the parameters of the student model, completing one iteration. This process is repeated multiple times during a training cycle. As the iterations progress, the model’s performance stabilizes, marking the completion of training. At the end of this process, the student model is employed for testing.

In the optimization process, the relative weighting of the classification and consistency losses dictates how supervised and unsupervised learning contribute to the model’s performance. The mathematical expressions for these classification and consistency losses are outlined as follows.

$$Classification\;Loss=w_{1}\sum_{i=1}^{L}y_{i}log\left(p_{s;i}\right)$$

$$Consistency\;Loss=w_{2}\sum_{i=1}^{L}{\left({p_{t;i}}^{⏝}p_{s;i}\right)}^{2}$$

In this project, weights $w_{1}$ and $w_{2}$ are assigned to the classification and consistency losses, respectively. The classification task involves three classes (L=3). The label for class *i* is denoted as $y_{i}$, while $p_{s;i}$ and $p_{t;i}$ are the probabilities predicted by the student and teacher models for class *i*. The weight for the consistency loss is calculated using the function $e^{-r{\left(1-x\right)}^{2}}*\left(w_{e}-w_{i}\right)+w_{i}$, where $x=\frac{ep}{eps}$, with *r* representing the coefficient for the consistency loss weight. The classification loss weight is determined by a function given by $1-\frac{ep-1}{eps}$, where ep represents the current epoch and eps is the total number of epochs. This function gradually decreases from 1 to 0 throughout the training period, allowing the model to initially converge quickly on labeled data and then progressively learn from unlabeled data.

The formula for Exponential Moving Average (EMA) is as follows:

$$\alpha_{t}=k\alpha_{t}+\left(1-k\right)\alpha_{s}$$

In this context, $\alpha_{t}$ and $\alpha_{s}$ represent the trainable parameters of the teacher and student models, respectively. The smoothing coefficient *k* is set to 0.98 throughout the training process.

**Figure 2:**
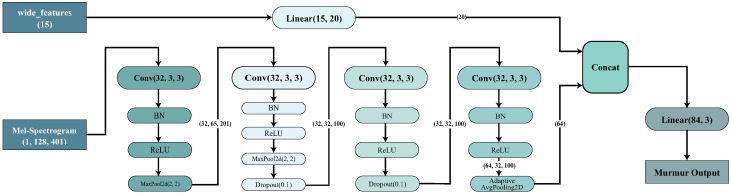
Architecture of the lightweight CNN model employed in mean teacher.

The following represents the foundational architecture of the teacher and student models, a lightweight CNN [4]; the architecture is shown in Figure 2. The lightweight CNN is the SOTA model in the Physionet 2022 challenge, outperforming other models due to its simple architecture, strong robustness, and generalization ability across different PCG signal data and populations. This makes it highly practical for use in PCG murmur detection.

This lightweight convolutional neural network architecture is specifically designed for processing audio data, particularly for identifying cardiac murmurs. The primary input to the model is a Mel-spectrogram with dimensions of (1, 128, 401), where 128 represents the frequency dimension and 401 represents the time dimension. Additionally, the model receives an auxiliary input called “wide features”, which is a 15-dimensional feature vector. This vector is processed through a linear layer to transform it into a 20-dimensional feature.

The model architecture consists of four convolutional blocks followed by a linear layer. Each convolutional block includes batch normalization and a ReLU activation function to enhance audio feature extraction. The first and second convolutional blocks have max-pooling layers with a (2, 2) window, which reduces the spatial dimensions of the feature maps and lessens the computational burden. Additionally, the second convolutional block features a dropout layer with a 0.1 dropout rate to mitigate overfitting. Following the convolutional blocks, an adaptive average pooling layer is used to further condense the feature space before passing it to the linear layer.

Finally, the model combines 20-dimensional “wide features” processed through a linear layer with the 64-dimensional features derived from the Mel-spectrogram via the adaptive average pooling layer, resulting in an 84-dimensional feature vector. This vector is then passed through another linear layer, which maps it to one of three output categories: “Present”, “Absent”, or “Unknown”. The design of the entire model aims to optimize audio classification accuracy by learning the intrinsic features of the Mel-spectrogram and the directly inputted “wide features”. Additionally, the simplified network structure maintains computational efficiency.

## 4. Experiment

The development environment for our project was built using Python 3.9.0, Nvidia A100 GPUs, Pytorch 2.1.0, and CUDA 12.1.0. For the training process, we implemented the ADAM optimizer, setting the learning rate to 1e-3 and using a batch size of 16. The detailed explanation is shown in Table 1.

### 4.1. Dataset and Preprocessing

In our research, we primarily utilized data from the George B. Moody PhysioNet Challenge for the years 2016 and 2022. The 2022 dataset comprises heart sound recordings from 874 patients, totaling 2982 entries collected from various auscultation points. Additionally, this dataset includes annotated patient information such as age, sex, height, weight, and other relevant variables. The recordings are categorized into three main groups based on the presence of murmurs in the PCG signals: Present, Absent, and Unknown. These categories serve as the labeled data for the model training. The 2016 dataset, designed for normal/abnormal classification rather than murmur detection, includes the 2016-e datasets renowned for their high-quality 2054 PCG recordings, which we used as unlabeled data in our analysis.

In our project, we standardized the duration of audio recordings, which originally ranged from 4.8 s to 120 s, by setting a fixed duration of 10 s for each recording. During preprocessing, recordings longer than 10 s were randomly cropped, while shorter recordings were padded with zeros to achieve a consistent length. This preprocessing method was uniformly applied during both the training and validation phases.

**Figure 3:**
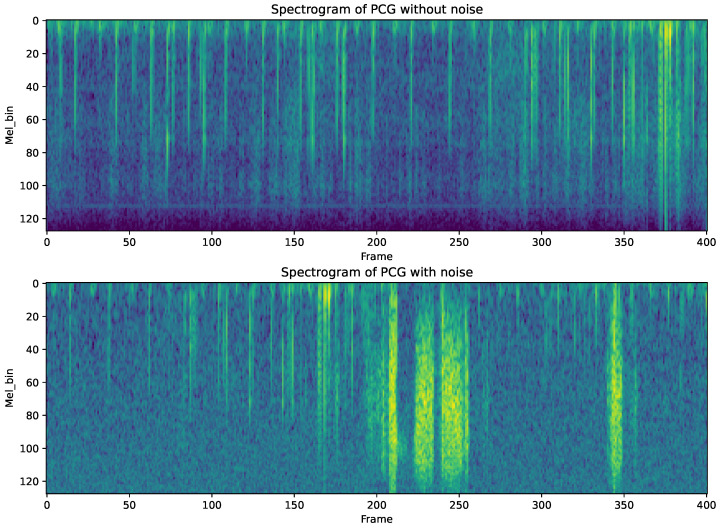
(**Top**): Mel-spectrogram of original PCG, (**Bottom**): Mel-spectrogram of PCG with Noise.

For each recording, we extracted a Mel-spectrogram with 128 Mel bands, using a window size of 50 ms and a frame shift of 25 ms, covering a frequency range from 25 to 2000 Hz, as illustrated in Figure 3. In addition to spectrogram extraction, we computed wide features, which were then categorized and encoded into one-hot vectors. To further enrich the dataset, we extracted statistical features from the audio signals, including the zero-crossing rate, spectral centroids, and spectral bandwidth.

**Figure 4:**
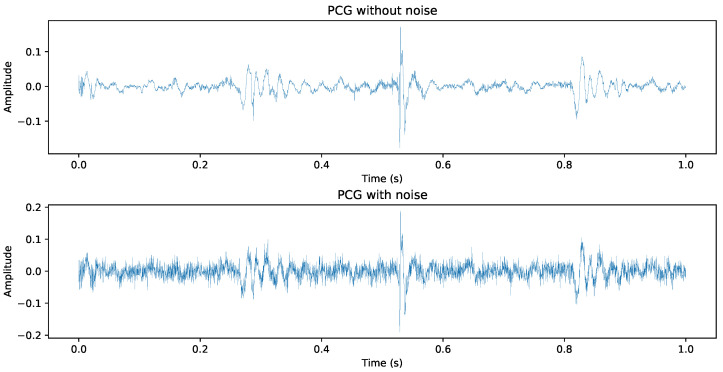
**Top**: Original PCG recording; **Bottom**: PCG recording after adding Gaussian noise.

To enhance the robustness of our model against overfitting and to simulate real-world noisy environments, we implemented two primary data augmentation techniques on the Mel-spectrograms. Initially, Gaussian noise with a zero mean and a standard deviation of 15 dB was added to each recording during training, as shown in Figure 4, with a probability of 0.5 for each signal. Additionally, we applied masking in both frequency and time domains to the spectrograms, as shown in Figure 3. This involved selectively blocking out multiple blocks of frequency channels and time steps. These augmentation strategies were designed to introduce variability and improve the generalizability of our model across diverse scenarios.

### 4.2. Results

Due to significant class imbalance in the dataset, we used the murmur score from the PhysioNet Challenge 2022 [9] and AUC indicators to provide an unbiased evaluation of our model’s performance. The murmur score is a weighted accuracy and can be defined by the confusion matrix shown in Table 2 and Formulation (4).

$$s_{m}=\frac{5m_{PP}+3m_{UU}+m_{AA}}{5\left(m_{PP}+m_{UP}+m_{AP}\right)+3\left(m_{PU}+m_{UU}+m_{AU}\right)+\left(m_{PA}+m_{UA}+m_{AA}\right)}$$

To demonstrate the robustness and practicality of our methodology, we refer to Table 3, which presents both the average values and their corresponding standard deviations. Initially, the 2022 dataset was segmented into five folds, ensuring that all recordings from the same subject were confined to a single fold. This subject-wise cross-validation approach guaranteed that no recordings from the same patient appeared simultaneously in both the training and validation/test sets, thereby mitigating the risk of data leakage and providing a more reliable evaluation of the model performance. Using one fold for labeled training and another for validation, this approach achieved a five-fold cross-validation murmur score of 0.7020 and an AUC of 0.7951. Subsequently, applying our mean teacher model, we designated one fold labeled as training data while gradually increasing the unlabeled data from one fold to three folds, with the remaining fold reserved for validation. This semi-supervised strategy improved the model’s performance, achieving a murmur score of 0.7540 and an AUC of 0.8562. As expected, we observed that as the amount of unlabeled data increased, the model’s performance improved accordingly. The *t*-test show a *t*-value of −18.88 leading to a *p*-value of 1.36 × 10^−4^ on Murmur Score, and a *t*-value of −99.55 leading to a *p*-value of 7.79 × 10^−8^ on AUC. These results indicate that incorporating an additional 60% of unlabeled data significantly improved the model’s performance over the baseline.

Furthermore, we conducted an experiment using a five-fold cross-validation approach, where four folds were used as labeled data and the fifth fold served as the validation set. This setup resulted in a murmur score of 0.8074 and an AUC of 0.8936, highlighting the effectiveness and robustness of the method, and establishing it as the current SOTA approach in this domain.

**Figure 5:**
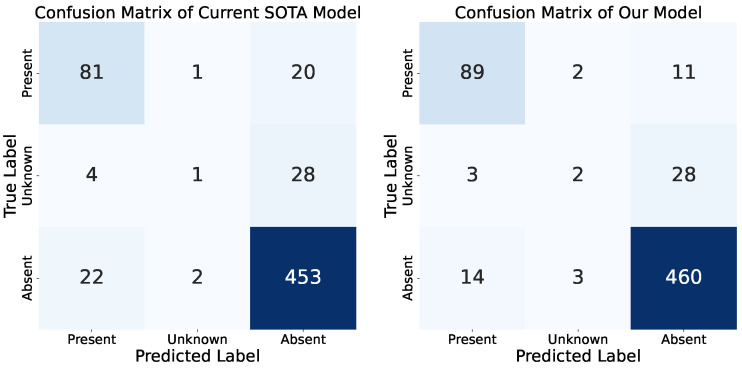
Comparison of the confusion matrix between the current SOTA model and our model.

To further enhance our semi-supervised learning strategy, we extended our approach by incorporating both the Physionet 2016 and 2022 challenge datasets. Specifically, we utilized 80% of the 2022 dataset as labeled data while using the entire 2016-e dataset as unlabeled data. The remaining 20% of the 2022 dataset was reserved for validation. This comprehensive configuration led to an improved murmur score of 0.8180 and an AUC of 0.9004. As shown in Figure 5, our model performs well in detecting murmurs in both the “absent” and “present” classes. However, it struggles significantly with the “unknown” class, which is the minority class in our dataset. This poor performance can also be attributed to the inherent clinical difficulty of the “unknown” class, even for clinical experts. Compared to the current SOTA model, our model significantly outperformed in both the present and absent class classifications.

These outcomes clearly demonstrate that our mean teacher method not only delivers more robust performance but also surpasses the results obtained by solely utilizing the 2022 challenge data. This underscores the effectiveness of incorporating unlabeled data to achieve a superior overall performance, highlighting the potential of our approach in advancing murmur detection and enhancing clinical decision-making in cardiovascular health. Consequently, our method establishes a new SOTA model for PCG murmur detection.

## 5. Discussion

While our research demonstrates significant advancements in PCG murmur detection using a semi-supervised mean teacher method, certain limitations persist. Typically, semi-supervised learning frameworks are most effective when the volume of unlabeled data substantially exceeds that of the labeled data. In our study, the volume of unlabeled data was comparable to that of the labeled data, potentially capping the performance improvements that could be achieved. This suggests that acquiring a larger pool of unlabeled data could further enhance the model performance by better leveraging the strengths of semi-supervised learning. In our preprocessing strategy, recordings longer than 10 s were randomly cropped. Incorporating all available 10-second segments and applying additional data augmentation techniques could help to expand the dataset and improve performance. Future efforts could also focus on expanding the dataset, particularly with high-quality unlabeled PCG recordings, to fully exploit the potential of our semi-supervised approach and push the boundaries of the current SOTA method.

Additionally, the Physionet Challenge 2022 and 2016 datasets may have certain flaws that need to be addressed. Although expert labels are always considered the ground truths in our training and validation processes, emerging research has indicated that relabeling can significantly improve model performance [36]. This insight suggests that expert labels, while highly reliable, may still contain some inaccuracies that can impact the model’s efficacy. A potential solution to this issue is to pinpoint ambiguous recordings within the dataset using the model’s initial outputs. Once these ambiguous cases are identified, they can be relabeled with the assistance of expert knowledge. By double-checking and refining the labels, this approach can help to eliminate any discrepancies and improve the overall quality of the dataset. Besides, previous studies on the Physionet Challenge 2022 primarily concentrated on the performance of the murmur score [4,5,6,26]. While we have incorporated AUC performance in our experimental evaluation, other metrics such as sensitivity and specificity could also be considered for a more comprehensive assessment. Moreover, using additional high-quality PCG datasets, even simultaneous PCG and ECG (Electrocardiogram) dataset [37] for multi-signal source training may further elaborate our model. Finally, traditional murmur detection systems focus primarily on identifying the presence or absence of murmurs without differentiating their underlying cause or severity. However, heart murmurs, while indicative of abnormal heart activity, can stem from a variety of causes. Some of them are benign and others associated with serious valvular heart diseases such as aortic stenosis or mitral regurgitation. It is crucial clinically to distinguish between murmurs that are significant and those that are less concerning. As such, enhancing murmur detection algorithms to automatically classify murmurs based on their clinical relevance would also mark a significant step forward.

Also, the model’s application to pediatric heart murmur detection and in LMICs presents certain limitations. As noted by [38], detecting heart murmurs in neonates and infants poses unique challenges due to the lower amplitude and higher variability of their heart sounds. Even though the PhysioNet2022 data focus on patients under 21 years old [9,39], for younger patients and children, the change in PCG signals would post challenge on our model. In addition, noisy environments are common in low-resource settings, where medical equipment may not meet optimal standards, can further degrade the model performance. The significant GPU computational requirements of the current model may also be difficult to meet in such environments. To address these challenges, future improvements should focus on creating pediatric-specific heart murmur datasets, enhancing the model’s robustness to noise, and reducing its hardware and computational demands.

## 6. Conclusions

Our study showcases the mean teacher model in PCG signal murmur detection, achieving SOTA performance. By integrating both labeled and unlabeled data from the Physionet 2022 and 2016 challenges, we significantly enhanced detection accuracy, culminating in an impressive murmur score of 0.8180 and an AUC of 0.9004. These results not only affirm our model’s superiority over existing methods, but also demonstrate its effectiveness in managing the sparse datasets typical in medical applications. The study underscores the critical role of extending the dataset with additional unlabeled data, which is pivotal in advancing semi-supervised learning to achieve better performance and robustness in clinical diagnostics. Furthermore, our method provides an accessible, highly accurate, and cost-effective approach for early-stage CVD diagnosis, especially in LMICs, aiming to improve clinical equity worldwide.

## Figures and Tables

**Table 1:** Key parameter configuration.

Parameter Name	Parameter Value	Parameter Description
Optimizer	Adam	Neural Network optimizer
Learning Rate	1 × 10^−3^	learning rate for optimizer
Batch Size	16	Number of samples per iteration.
EMA k	0.98	Smoothing coefficient in Exponential Moving Average.
Weight con init	0.7	Initial value for consistency loss in mean teacher.
Weight con end	1	Final value for consistency loss in mean teacher.

**Table 2:** Confusion matrix for murmur detection.

	Murmur Expert			
Murmur Classifier		Present	Unknown	Absent
	Present	$m_{PP}$	$m_{PU}$	$m_{PA}$
	Unknown	$m_{UP}$	$m_{UU}$	$m_{UA}$
	Absent	$m_{AP}$	$m_{AU}$	$m_{AA}$

**Table 3:** Comparative analysis of various training modes across different data splits.

	Data Split			Result Measurements	
**Model**	**Labeled Data**	**Unlabeled Data**	**Validation Data**	**Murmur Score**	**AUC**
Full Supervise	20% v2022	/	20% v2022	0.7020 ± 0.0026	0.7951 ± 0.0008
Semi Supervise	20% v2022	20% v2022	20% v2022	0.7298 ± 0.0035	0.8217 ± 0.0009
Semi Supervise	20% v2022	40% v2022	20% v2022	0.7418 ± 0.0021	0.8398 ± 0.0013
Semi Supervise	20% v2022	60% v2022	20% v2022	0.7540 ± 0.0040	0.8562 ± 0.0007
Full Supervise **(Current SOTA)**	80% v2022	/	20% v2022	0.8074 ± 0.0010	0.8936 ± 0.0011
Semi Supervise **(Ours)**	80% v2022	100% v2016-e	20% v2022	**0.8180 ± 0.0006**	**0.9004 ± 0.0019**

## Data Availability

The original data used in this study are openly available at https://physionet.org/content/challenge-2016/1.0.0/ and https://physionet.org/content/challenge-2022/1.0.0/. The models presented in this study are available upon request from the corresponding author.
